# Membrane Partitioning of TEMPO Discriminates Human Lung Cancer from Neighboring Normal Cells

**DOI:** 10.32607/actanaturae.19426

**Published:** 2023

**Authors:** O. K. Gasymov, M. J. Bakhishova, R. B. Aslanov, L. A. Melikova, J. A. Aliyev

**Affiliations:** Institute of Biophysics, Ministry of Science and Education Republic of Azerbaijan, Baku, AZ1171 Azerbaijan; National Center of Oncology, Azerbaijan Republic Ministry of Health, Baku, AZ1012 Azerbaijan

**Keywords:** electron paramagnetic resonance, TEMPO partitioning, lung carcinoma, cell membrane lipid composition, cell membrane sensitivity

## Abstract

The plasma membranes of normal and cancer cells of the lung, breast, and colon tissues show considerably different lipid compositions that greatly influence their physicochemical properties. Partitioning of the spin probe 2,2,6,6-tetramethylpiperidine-1-oxyl (TEMPO) into the membranes of human lung normal and carcinoma cells was assessed by EPR spectroscopy to estimate the impact of the lipid compositions. The goal was to reveal potential strategies for cancer therapy attributable to the membrane properties. The study was conducted at pH values of 7.3 and 6.2, relevant to the microenvironments of normal and cancer cells, respectively. The TEMPO partitioning was examined in the temperature interval of 283–317K to reveal the efficacy of local hyperthermia used in chemotherapy. Results indicate that the TEMPO partitioning coefficient for the membranes of human lung carcinoma cells is significantly higher compared with that of neighboring normal cells. Increased partition coefficients were observed at relatively higher temperatures in both normal and cancer cells. However, compared to the normal cells, the cancer cells demonstrated higher partition coefficients in the studied temperature range. The data obtained with C12SL (spin-labeled analog of lauric acid) indicate that increased membrane dynamics of the cancer cells is a possible mechanism for enhanced partitioning of TEMPO. Free energy values for partitioning estimated for pH values of 6.2 and 7.3 show that TEMPO partitioning requires 30% less energy in the cancer cells at pH 7.3. TEMPO and its derivatives have previously been considered as theranostic agents in cancer research. Data suggest that TEMPO derivatives could be used to test if complementary alkalization therapy is effective for cancer patients receiving standard chemotherapy with local hyperthermia.

## INTRODUCTION

Cancer cells, even within the same tumor mass, show heterogeneity in both the phenotypic and functional levels. The heterogeneity of the cancer cell population is dynamic and susceptible to significant modifications by various factors during cancer development [1, 2]. In the course of development, cancer cells acquire new capabilities, such as evading apoptosis, self-sufficiency in growth signals, insensitivity to anti-growth signals, tissue invasion and metastasis, limitless replicative potential, and sustained angiogenesis. It is contended that these capabilities are shared in all types of human tumors [3]. Metastasis of cancer cells is the major cause of mortality in cancer patients. Epithelialmesenchymal transition in cancer cells (carcinomas) is critical for the development of metastasis capability. Several steps are involved in metastatic progression, during which cancer cells lose their polarity, cell-tocell adhesion, etc. All these changes are manifested in the cell membranes that play a fundamental role in cell functioning [3, 4].

One of the main components of a cell membrane is a lipid bilayer that contains various lipids, such as asymmetrically arranged phospholipids, sphingolipids, glycolipids, cholesterol, etc. [5, 6, 7]. A wide variety of proteins, called transmembrane proteins, are embedded in the cell membranes and protrude on one or both sides. There are also peripheral membrane proteins that temporarily associate with the membranes of the cells to perform various functions. Both membrane-embedded and -associated proteins and peptides play a critical role in cell functioning, particularly in cellular signal transduction. Often, for the cells to execute specific functions, the actions of these proteins need to be regulated in an orchestrated manner [4, 8, 9, 10]. Most studies related to cell membrane functions are devoted to investigating the proteins involved in various signaling pathways [11]. However, the lipid compositions provide not only specific hydrophobic environments for the proper folding of the membrane proteins, but also modulate their functions and participate in the maintenance of cell architecture [5, 6, 7]. Yet, relatively little attention has been paid to the functional role of lipids and lipid domains in the cell membrane.

A large body of evidence has been accumulated that supports the critical role of lipid compositions in healthy cell membranes and their significant alterations in various diseases, including cancer [12, 13, 14].

Lipid compositions play a pivotal role in cell functioning. Based on this observation, modulation of cell membrane components and/or properties has been proposed as a new therapeutic strategy for cancer therapy [13]. Lateral arrangements of the lipids in the membranes of the cells are heterogeneous and described as membrane lipid domains [15, 16, 17, 18]. The lipid domains composed of various types of lipids are functional as lipids but indirectly can also influence and/or modulate membrane function. The specific composition of each lipid domain determines its distinct physicochemical properties [12, 14]. The lipid compositions of the membranes of cancer cells are significantly altered compared to those of healthy controls [13, 14, 19]. These findings provide the basis to characterize cancer cells by studying the lipid micro-environment of the membranes.

Lipid reprogramming of cancer cells and their possible mechanisms of action have also been investigated for lung cancer, particularly in non-small cell lung cancer (NSCLC). Lipid composition is also pivotal for NSCLC cell migration. It has been shown that migration of these cells can be inhibited considerably by cholesterol depletion in lipid rafts [20]. Progression of many types of cancer cells, including NSCLC, requires altered and enhanced fatty acid metabolism to support cell division and growth. In preclinical models, inhibition of acetyl-CoA carboxylase, the enzyme that regulates *de-novo *fatty acid synthesis, represses tumor growth in NSCLC [21, 22].

Overexpression of fatty acid synthase (FAS), a lipogenic enzyme, is observed in various types of cancer, including lung, colon, and prostate cancers. FAS provides a *de-novo *fatty acid synthesis that modifies the lipid compositions of cancer cells [23]. Stearoyl-CoA desaturase 1 (SCD1) is another protein involved in lipid metabolism that plays an essential role in the malignant transformation of lung cancer cells [24, 25]. Desaturation and prolongation of fatty acids have been shown for lung cancer cells. In the desaturation event, each double bond in the *cis *configuration creates a twist in the acyl chain that, in turn, increases the membrane fluidity. Increased membrane fluidity induced by desaturation stimulates cancer metastasis and is associated with poor prognosis in lung cancer patients.

Electron Paramagnetic Resonance spectroscopy (EPR) with the use of various nitroxide probes has been developed as a powerful tool to characterize the lipid micro-environment of the cell membranes. Characterization of the lipid micro-environments of the cell membranes of healthy and cancer tissues is important to understand the functional changes in the cancer cell membranes associated with lipid components. The sensitivity of cancer cell membranes to relevant environmental conditions is an important attribute in developing a method for preferential drug delivery to cancer cells using the differences in the properties of the lipid domains. Previously, to segregate the contribution of only lipid components of the cell membranes, we investigated the properties of liposomes fabricated using lipids extracted from human lung cancer and normal cells [26, 27]. The liposomes composed of the cancer cell lipids showed significantly enhanced partitioning of spin probe 2,2,6,6-tetramethylpiperidine-1-oxyl (TEMPO) compared to those fabricated using normal cell lipids. In the current study, the partitioning of TEMPO into the membranes of live cells of human lung normal and carcinoma tissues was examined. A wide assortment of nitroxide spin probes can be used to characterize different regions of the cell membrane. TEMPO, used in this study, does not show any affinity to the membrane proteins and, therefore, provides a characterization of the lipid phase of the membrane as a separate component. However, unlike the studies performed in liposomes, the lipid phase of the membrane is modified by the presence of membrane proteins. The experiments were performed in a temperature interval of 283–317 K, the highest value of which matches the condition used in local hyperthermia [28, 29]. The experiments at pH 6.2 mimic the acidic environment created in cancer development [30, 31, 32]. The study revealed differences in parameters (polarity, micro-viscosity, and the energy required to transfer TEMPO from the aqueous to the membrane environment) between the membranes of cancer and healthy cells. Compared with previous works on liposomes [26, 27], the results obtained in this study indicate that pro teins embedded in the cell membranes significantly alter the dynamics of the lipid fraction, making them more dynamic and permeable to small molecules. The determined temperature and pH sensitivities of the cell membranes may help to choose or create the appropriate conditions for cancer therapy.

## MATERIAL AND METHODS

**Human lung tissue collection**

**Fig. 1:**
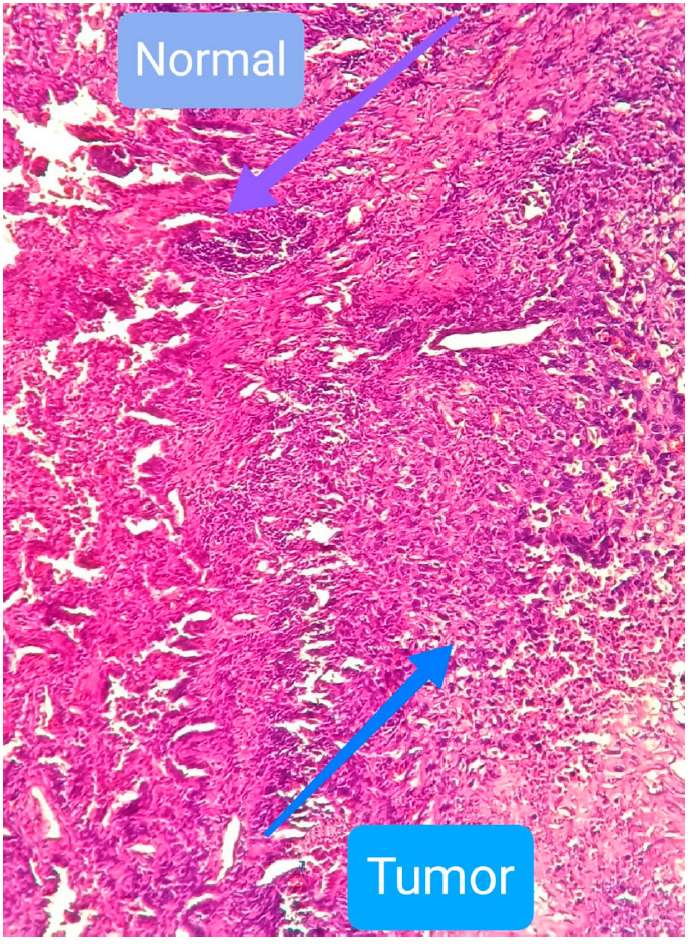
Normal and cancer cells in lung tissue

Human lung tissues were collected immediately after the surgery on lung carcinoma patients in accordance with the tenets of the Declaration of Helsinki and approved by the review board of the Azerbaijan National Center of Oncology. Informed consent was obtained from each donor. Lung carcinoma patients were selected after computed tomography. The cancer diagnosis for individuals was confirmed after biopsy and subsequent histopathological grade (aggressiveness) evaluations. Experiments were performed on five individuals. However, due to similar findings, here we report, as an example, a case of a 52-year-old male who did not receive chemo- or radiation therapy before the surgery. The results for this case were more characteristic and, therefore, analyzed thoroughly. The pathology findings on the surgical lung tissue were consistent with Stage II, pT2bN0Mx, non-small cell lung cancer. Bulk lung tissue was segregated into cancer (carcinoma) and neighboring normal (also referred to as healthy) tissues by the pathologist. Normal and cancer cells in the investigated lung tissue are indicated in *Fig. 1*. Experiments with spin-labeled lauric acid (C12SL) were performed with the surgical tissue of the patient with the following pathology findings: 53-years-old male, lung adenocarcinoma, Stage II, pT- 3N0Mx.ICD-O: 8260/3, invasive.

**Preparation of epithelial cell suspension from lung tissue**

The fresh lung tissue (about 2-3 h after the surgery) was washed thoroughly with PBS buffer to remove blood. Afterward, the tissue was cut into small pieces and then homogenized in PBS buffer using a glass homogenizer. The homogenized lung tissue was washed three times with PBS solution and then centrifuged (Eppendorf 5418) to remove the cell debris. Obtained cell suspension was used for the experiments. The intactness of the cells was assessed by Zeta-potential measurements as shown previously [33].

**EPR spectroscopy**

EPR measurements were performed using a Bruker ELEXSYS E580 spectrometer at X-band frequency with variable temperature accessory. The aqueous suspension of lung carcinoma and neighboring normal cells with TEMPO were placed into Pyrex capillary tubes with an I.D. of 0.6 mm. EPR spectra were recorded with the following instrument parameters: scan width: 100 Gauss; sweep time: 40 s; modulation amplitude: 1 Gauss; modulation frequency: 100 kHz; microwave power: 0.47 mW; and time constant: 0.1 s. Before the measurements, the samples were kept for 5 minutes at each temperature to ensure that the sample temperature matched the set temperatures.

**Partitioning of TEMPO in the membranes of human lung normal and carcinoma cells**

TEMPO dissolved in an aqueous solution displays a well-known EPR spectrum with three components resulting from nitrogen hyperfine interactions. However, TEMPO incubated in the cell environment shows a composite EPR spectrum, the third component (located in a high magnetic field) of which is partially resolved. The difference in the nitrogen splitting constant of TEMPO in hydrophobic (cell membrane) and hydrophilic (aqueous) environments is the reason for the split of the third component. Consequently, the EPR spectra of TEMPO incubated with cells in an aqueous environment reflect the partitioning of TEMPO in the lipid fraction of the cell membrane and aqueous environments. To resolve the spectral components, the EPR spectra of TEMPO were analyzed using the LabVIEW program developed by Christian Altenbach (https://sites.google.com/site/altenbach/), using the spectral simulation code written in FORTRAN [34]. Along with computer simulations, rotational correlation times of TEMPO were also calculated using the following formula that uses the peak heights and line widths of the first derivative EPR spectra [35]:

τ_c_ = 6.5 × 10^-10^*W*_0_ [(*h*_0_ /*h*_-1_)^1/2^ -1],

where *W*_0_ is the width (in Gauss) of the central component, while *h*_0_ and *h*_-1_ are the heights of the central and high-magnetic field components of the first derivative EPR spectrum. As mentioned above, the EPR spectra resulting from the partitioning of TEMPO in the system are composite and consist of two components. Therefore, the formula above was applied after the decomposition of the EPR spectra into lipophilic and hydrophilic components. Because of the close similarity of the correlation times obtained from the software and the formula, data are shown only as deduced from the software.

Double integrals of the EPR spectral components (*I_mbr_*and *I_aq_*represent TEMPO confined in the membrane and aqueous environments, respectively) were employed to calculate partition coefficients with the following formula: *K *= *I_mbr_*/(*I_mbr_*+ *I_aq_*). To characterize the EPR spectral components, the membrane and aqueous environments were described as lipophylic and hydrophilic, respectively. Apparently, a partition coefficient depends on both the concentration of the lipid fractions and the number of lung cells in the aqueous system [36]. For an accurate comparison of the data related to the partition coefficients, the same amount (by weight) of cancer and normal cell suspensions were used. Both cancer and normal cell suspensions were incubated with TEMPO (150 mM total concentration) for about 30 min. The experimental conditions used in this study allow us to compare the partition coefficients of the normal and cancer cells directly.

The temperature dependence of the equilibrium constant *K *(partition coefficient in our case) was used to calculate the free energy changes required to transfer TEMPO molecules from the aqueous to the lipid phase of the membranes of the healthy and cancer cells.

log*K *= −∆*G*/*RT*

Similarly, the temperature dependence of the rotational correlation times of TEMPO was used to determine the activation energies for rotational motions in the membranes of the healthy and cancer cells.

**Dynamics of the lipid domain of the cell membrane evaluated by spin-labeled lauric acid analog (C12SL)**

Experimental procedures with C12SL, the chemical structure of which is shown below in the relevant* Figure*, were similar to that of TEMPO described in section 2.4. In contrast to TEMPO, C12SL was dissolved in ethanol. An equal amount of each cell suspension (0.1 mg/ml) was incubated with 200 mM of C12SL for 30 min. A high concentration of C12SL was employed to monitor both the dynamics of the lipid domain and its maximal incorporation capacity in healthy and cancer cells. The attained complex EPR spectra were analyzed with a multi-component EPR program [34]. Analyses of the EPR spectra of C12SL were performed in two steps. In the first step, free C12SL, not incorporated into the cell membrane, was removed to decrease the number of fitting parameters. Removal of free C12SL spectra was performed using the EPR program “FreeRemover”, which is part of the program package [34]. In the second step, the spectra that lack “free” spectral components were analyzed by the multi-component EPR spectral analysis as described above.

## RESULTS AND DISCUSSION

As indicated above, the lipid compositions in the healthy and cancer cells of various tissues significantly differ from each other [12, 13, 14]. The lipid compositions of the cells determine the specific properties of the cell membranes, such as membrane fluidity, permeability, the temperature of phase transition, etc. These characteristics of the membranes are essential from a therapeutic point of view, particularly for drug delivery applications. TEMPO does not show any binding properties toward the proteins. Therefore, the use of TEMPO in membrane research allows for a selective characterization of the lipid phase. Unlike the findings obtained in liposomes [26, 27], in this case, lipid phase properties are modified by the membrane proteins of the corresponding cells. Below, we provide experiments of TEMPO partitioning into the membranes of the cells at the temperature of 283–317 K interval and pH values of 7.3 and 6.2, conditions that are relevant for cancer therapeutics.

**Partitioning of TEMPO into the membranes of healthy and cancer human lung cells**

**Fig. 2:**
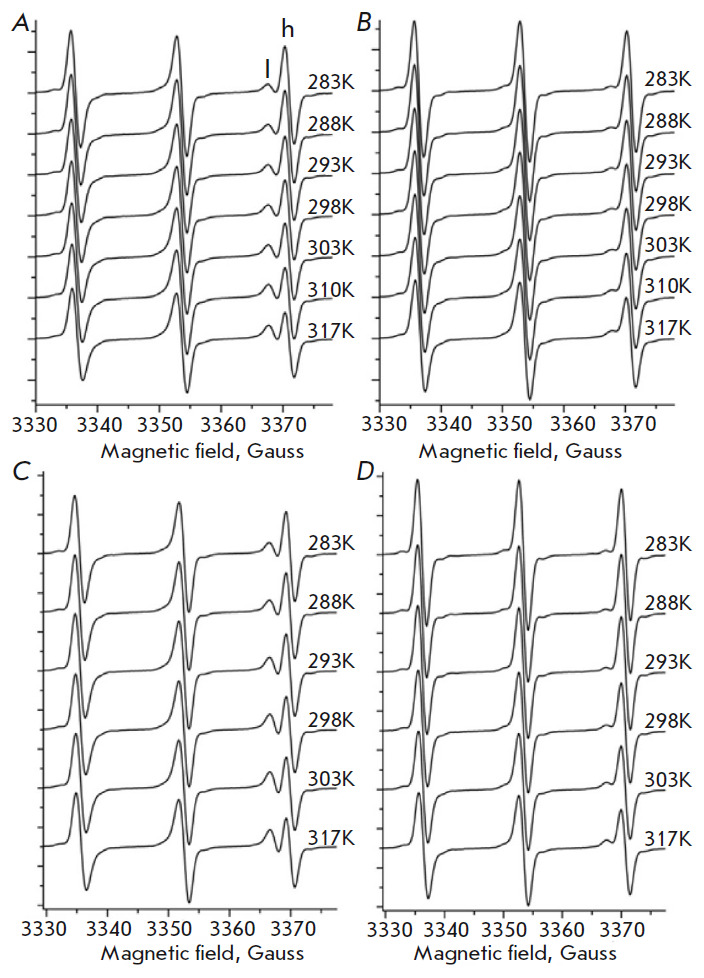
The...

The EPR spectra of TEMPO incubated with human lung cancer and healthy cells at pH 7.3 are shown spectra in the high magnetic field split into two peaks labeled as l (lipophylic) and h (hydrophilic). Thus, each EPR spectrum is composed of two components resulting from the partitioning of TEMPO between the cell membrane (lipophylic) and aqueous (hydrophilic) phases. The relative amplitudes of the high-field components of EPR spectra indicate that the partition of TEMPO is significantly different for cancer and healthy cells (*Fig. 2*). At pH 7.3, the relative amount of TEMPO in the membranes of the cancer cells is significantly higher compared to that in healthy cells. Interestingly, differences in TEMPO partitioning are even higher at pH 6.2 compared to those at pH 7.3 (*Fig. 2A,C*). EPR spectra in the temperature interval of 283−317 K indicate that an increase in temperature further augments the relative amount of TEMPO in the membranes of both cell types. However, compared to healthy cells, the membranes of cancer cells incorporate more TEMPO molecules in the respective conditions.

To describe the EPR spectra of TEMPO in environments with significantly different hydrophobicity values, one should consider the following aspects. The observed splits of the EPR components in a high magnetic field arise from the small differences in the isotropic hyperfine coupling constants (Aiso) and *g *factors of the nitroxide spin probe in each environment. These differences are explained as changes in the relative contribution of two canonical structures of TEMPO as shown below [37].

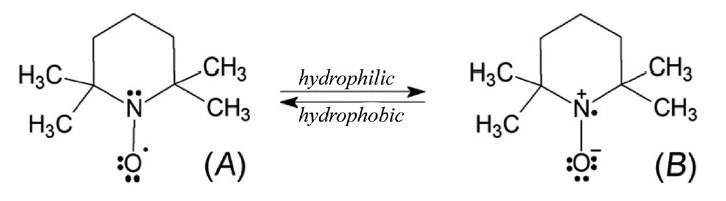



Polar solvents like the aqueous solution tend to stabilize the structure (*B*) in which the unpaired electron density is localized on the N-atom. The increased relative contribution of structure (*B*) gives rise to the nitrogen hyperfine coupling constant. In contrast, structure (*A*), in which the unpaired electron density is localized on the oxygen atom, is preferentially stabilized in a hydrophobic environment. Therefore, the nitrogen hyperfine coupling constant is lower in a hydrophobic environment compared to that of a hydrophilic environment.

**Fig. 3:**
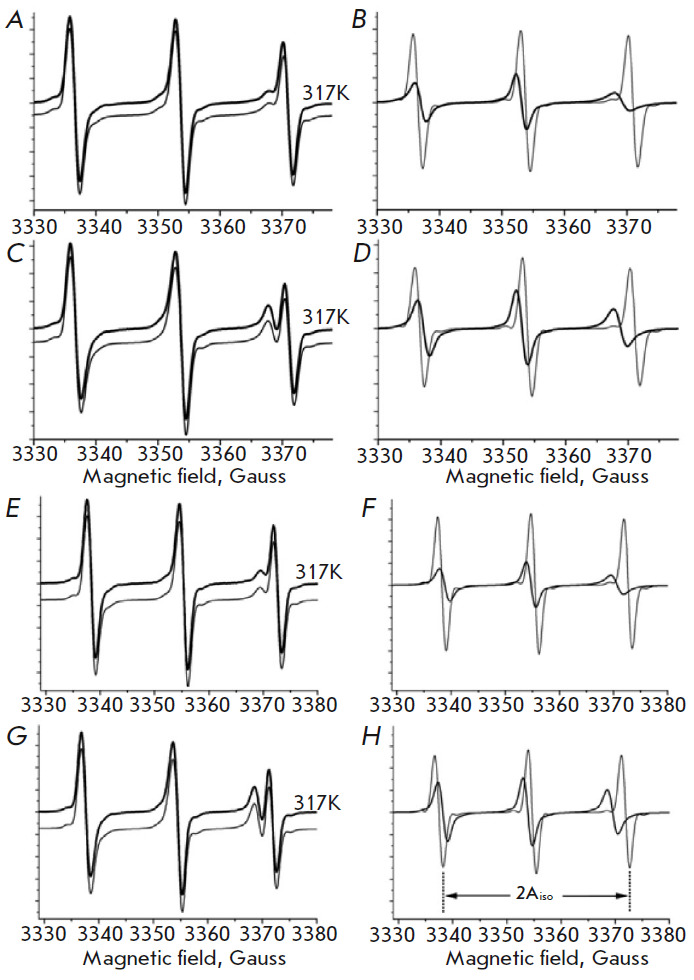
The...

However, the 2Aiso values of TEMPO incorporated into the membranes of the cells are significantly decreased and fall within an interval of 31.4−32.4 Gauss. At pH 7.3, 2Aiso values for the EPR spectra of TEMPO incorporated in the membrane of healthy and cancer cells are 32.4 Gauss and 31.5 Gauss, respectively. Thus, the lipid fractions of the cancer cell membrane are more hydrophobic compared to those of the healthy cells. Interestingly, as judged from the Aiso values of the EPR spectra of TEMPO, when increasing the pH from 6.2 to 7.3, a small increase in hydrophobicity was observed in the membranes of healthy cells (from 31.9 Gauss to 32.4 Gauss) but not in cancer cells (about 31.5 Gauss). As a result, at pH 6.2 the difference in hydrophobicity values is shrunken between healthy and cancer cell membranes.

**Table 1:** Parameters obtained from the EPR studies on the membranes of healthy and cancer cells of the human lung

Sample	2A_iso_, Gauss		DG_t_	DG_K_
	aqueous	membrane	kcal/mol	
Healthy cells, pH 7.3	34.5	32.4 1.9 ± 0.2	3.1 ± 0.1	
Cancer cells, pH 7.3	34.5	31.5	2.1 ± 0.2	1.2 ± 0.1
Healthy cells, pH 6.2	34.4	31.9	3.8 ± 0.3	3.7 ± 0.3
Cancer cells, pH 6.2	34.5	31.4	2.6 ± 0.3	1.7 ± 0.1

TEMPO does not show any binding affinity toward proteins. Therefore, the EPR spectra of TEMPO assigned to the lipophilic phase displays the characteristics of the lipid fraction of the cell membranes. The partition coefficients for the cell membranes are significantly higher compared to those obtained in liposomes fabricated from the corresponding cells [26, 27]. Thus, the proteins embedded into the membranes modify lipid phase properties, resulting in augmented partitioning of TEMPO.

**Micro-viscosity of the lipid fraction of the cell membranes**

**Fig. 4:**
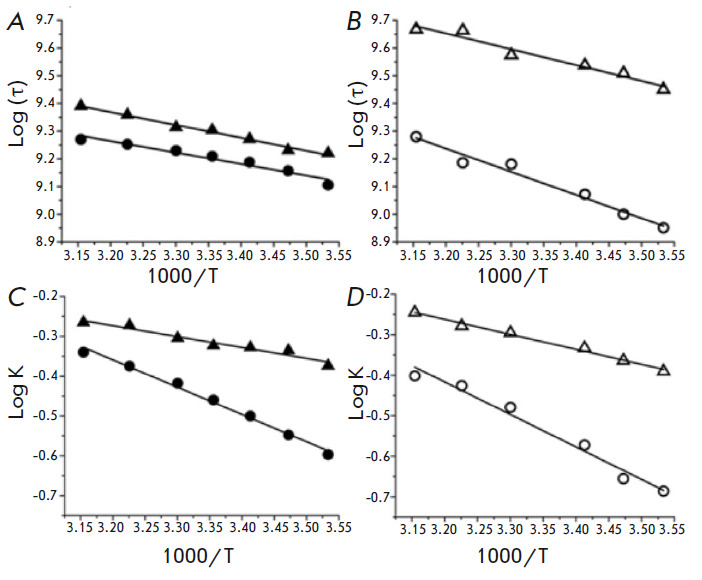
The...

Along with the decomposition of the EPR spectra of TEMPO, computer analysis also allows us to estimate the rotational correlation times of TEMPO corresponding to the spectral components in various environments. In lipid fractions, the rotational correlation times of TEMPO in cancer cells are decreased compared to those obtained in healthy cells at both pH values (7.3 and 6.2) and the used temperature range (*Fig. 4*). For example, at room temperature and pH 7.3, rotational correlation times are about 490 ps and 617 ps (*Fig. 4A*) for TEMPO incorporated into cancer and healthy cell membranes, respectively. Faster rotation (corresponding to a lower correlation time) of TEMPO indicates a low viscosity of the surroundings. Since TEMPO molecules reside in the lipid fraction, the data indicate enhanced dynamics of the lipid fraction.

The Arrhenius plots for the correlation times obtained at various temperatures are shown in *Fig. 4A,B*. The activation energies of the rotational motions of TEMPO (∆Gτ) in the membranes of healthy and cancer cells of the human lung at pH values of 7.3 and 6.2 are shown in *Table 1*. The values of ∆Gτ for the membranes of healthy and cancer cells are very similar (∆Gτ values are 1.9 ± 0.2 and 2.1 ± 0.2 kcal/mol, respectively), indicating the comparable micro-viscosities of the studied lipid fractions in both cases. At pH 6.2, increased values of ∆Gτ were observed for both cell types. However, the micro-viscosity of the membrane fractions of the healthy lung cells was higher compared to those of the cancer cells (∆Gτ values are 3.8 ± 0.3 and 2.6 ± 0.3 kcal/mol, respectively).

**Efficiency of the transfer of TEMPO molecules from an aqueous to a lipid phase of cell membranes**

Computer-assisted decompositions of the EPR spectra of TEMPO incubated with the healthy and cancer cells provided a means to conduct an evaluation of its partition coefficients. Several factors, such as lipid compositions, membrane dynamics, etc., may influence the partition of molecules (TEMPO in this study) between the membrane and the aqueous environment. Because TEMPO does not show any binding affinity toward proteins, equilibrium in the partitioning can be considered as a result of the passive incorporation of the molecules into the lipid fractions of the membranes. At this point, the term ‘passive incorporation’ indicates that the proteins localized on the cell membrane are not participating directly in this process. However, the membrane proteins may alter the properties of the lipid phase capable of influencing the partitioning characteristics of TEMPO in the cell membranes.

The Arrhenius plots for the partition coefficients (*K*) obtained at various temperatures are shown in *Fig. 4C,D*. The standard Gibbs free energy change required to transform a TEMPO from an aqueous to a lipid phase of the membrane of healthy and cancer cells is shown in *Table 1*. At pH 7.3, free energy changes for the transfer of TEMPO from aqueous phase to lipid phase in healthy and cancer cell membranes are 3.1 ± 0.1 kcal/mol and 1.2 ± 0.1 kcal/mol, respectively. In the acidic transition from pH 7.3 to pH 6.2, the free energy changes of TEMPO transfer for healthy and cancer cell membranes increase by about 19% and 42%, reaching the values of 3.7 ± 0.3 kcal/mol and 1.7 ± 0.1 kcal/mol, respectively. Thus, more energy is required to transfer TEMPO to the cell membranes in an acidic pH. Data indicate that in lung tissue composed of both healthy and cancer cells TEMPO molecules will preferentially incorporate the membranes of cancer cells.

It is well established that during the progression of the disease, the cancer cells in hypoxic conditions increase glucose consumption via aerobic glycolysis (termed as the Warburg effect). This process results in the creation of an acidic micro-environment [30, 31, 32, 38, 39, 40, 41]. Cancer cells effectively use the acidic micro-environment for mesenchymal transition and metastasis. If TEMPO is considered as a model for certain drugs, then for drug delivery, it would be beneficial to create normal pH (7.3) conditions for the cancer cells. Differences in the ∆G*K *values for healthy and cancer cells are almost identical for pH 7.3 and pH 6.2. However, the ∆G*K *value is at its smallest for cancer cells at pH 7.3, indicating that less energy is required to transfer TEMPO from the aqueous solution to the membrane. The current study has direct clinical value. TEMPO and its derivatives show significant anti-cancer effects when applied to various types of cancer, including lung cancer [42, 43, 44, 45, 46, 47]. It is highly anticipated that TEMPO-benzoate, which shows significantly enhanced partitioning in liposome studies [26, 27], will also be very effective in the membranes of the corresponding cells. A FTIR study of the lipids extracted from the normal and cancer cells supports this finding. In contrast to normal cells, the lipid fractions from the cancer cells are in a more disordered state. In addition, lipids from the cancer cells exhibit a non-cooperative temperature transition, as opposed to the cooperative temperature transition observed for the healthy cells. The results obtained from numerous human lung cancer samples will be published elsewhere.

**Evidence of increased dynamics of the lipid phase in the membranes of cancer cells compared to healthy cells**

**Fig. 5:**
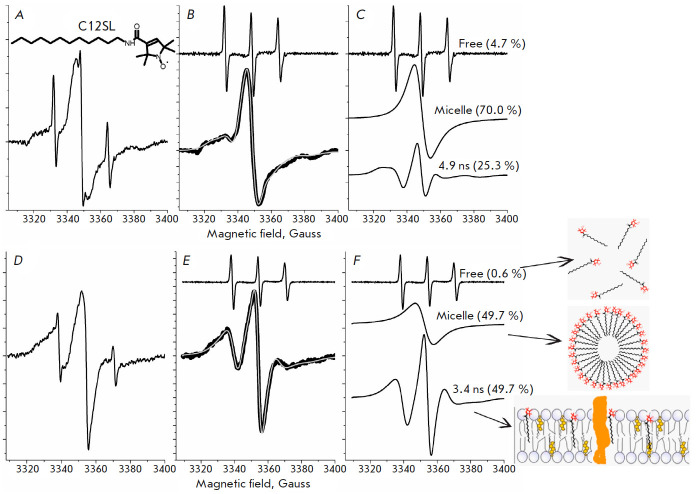
EPR spectra of C12SL incorporated into the lipid domain of the membranes of healthy and cancer cells of the human lung. (*A*), (*B*), and (*C*) are the EPR spectrum of C12SL (shown in (*A*)) incubated with the cell suspension of healthy lung tissue, separation of free and composite spectral components, and the spectral component of the EPR spectrum, respectively. The grey line (in (*B*)) is a computer-simulated spectrum from the best fit parameters of a two-component model. (*D*), (*E*), and (*F*) are the same as in (*A*), (*B*), and (*C*), but they were obtained from the cell suspension of lung cancer cells. The EPR spectra of C12SL were measured at room temperature. The schema provided in the bottom-right corner of the *Figure *illustrates C12SL in “free”, “micelle” and “membrane-incorporated” situations. C12SL is a spin-labeled analog of lauric acid, the chemical structure of which is shown in *Fig. 5A*

The dynamics of the lipid phase of the membranes of healthy and cancer cells were assessed using a C12SL (*Fig. 5*). C12SL molecules possessing an amphiphilic nature are predisposed to incorporate the lipid phase of the membranes. However, the efficiency of the incorporation depends on the physicochemical properties of the membranes, mainly based on fluidity (dynamics). Because of the position of the nitroxide spin label on C12SL, the dynamic parameters obtained from EPR spectra will be related to the surface part of the membranes [48].

The EPR spectrum of C12SL incorporated into the membranes of healthy lung cells (pH 7.3, room temperature) is shown in *Fig 5A*. The best fit spectra obtained from a computer analysis indicate that two components (besides the free components) are sufficient to describe the composite EPR spectra (*Fig. 5B,C*). About 25% of C12SL is incorporated into the membranes of healthy cells and its rotational correlation time amounts to about 4.9 ns. Because of limited solubility, about 70% of C12SL is in micelle form in aqueous environments. The broad singlet spectrum results from strong spin-spin exchange interactions where nitroxide spin labels are too close to each other. The EPR spectrum of C12SL incorporated into the membranes of the cancer cells of human lung tissue (*Fig. 5D*) is significantly different from those obtained from healthy cells (*Fig. 5A*). In contrast, about a twofold higher amount of C12SL was incorporated into the membranes of cancer cells of the lung. Besides that, the dynamics of the C12SL incorporated into cancer cell membranes are significantly increased, as is apparent by the decreased rotational correlation time (3.4 ns versus 4.9 ns). Data indicates that the membranes of cancer cells are more loosely packed than those of healthy cells, resulting in more permeability. Consistent with other findings, in the case of cancer cells, a lower fraction (50% versus 70%) of the C12SL is in aggregated form. Thus, the experimental data obtained with C12SL clearly indicate that, compared to healthy cells, the membranes of cancer cells are more dynamic.

**The cytotoxicity values (IC_50_) for TEMPO and its derivative compounds relevant to various applications**

Nitroxides and their different derivatives exhibit numerous biologically significant functions [43]. Therefore, different objectives have been considered in their applications to various diseases, including cancer therapy. The drug applications require a unique range of IC_50_ (the half-maximal inhibitory concentration) values. In cancer, nitroxides can be used as a radioprotector and contrast-enhancing agents in MRI (magnetic resonance imaging) [49]. For therapeutic applications, nitroxides possessing low cytotoxicity (therefore high IC_50_ values) are preferable. TEMPO and 4-hydroxy-TEMPO (aka TEMPOL), exhibiting IC_50_ values of 2.7 mM and 11.4 mM, respectively, are best suited for these proposes [50]. As an antiproliferative agent, the IC_50_ values of TEMPOL for various cell lines related to breast, colon, liver, and ovary cancer fall in the range of 0.21−1.073 mM [51]. TEMPOL provides a significant adjuvant effect in cancer applications. In some cell lines for colon cancer, TEMPOL significantly enhances the cytotoxicity of the widely used anti-cancer drug doxorubicin. In the HCT116 cell lines, pretreatment with TEMPOL shows about a 7-fold decreased IC_50_ value (from 0.38 mM to 0.053 mM). Some modified nitroxides show remarkable cytotoxicity against many cancer lines (IC_50_ values of about 0.06 μM, including A549 cells, which are the culprit cell lines for human lung cancer [52].

The current study is also relevant to cytotoxicity studies. It has been shown that nitroxide cytotoxicity is strongly related to the lipid/water partition coefficients [53, 54]. Indeed, as shown above, the IC_50_ value of the more lipophilic compound TEMPO is about 4-fold lower compared to that of the hydrophilic compound TEMPOL (just the –OH group attached to TEMPO). In line with these findings, benzoate group attachment to TEMPO dramatically enhances the partition coefficient in liposome studies [26, 27]. Thus, depending on the specific task at hand, the cytotoxicity of nitroxides (IC_50_ values) can be considerably modified by an assortment of group attachments. The membrane partitioning values determined by the use of EPR spectroscopy can provide a preliminary, quick assessment of the cytotoxicity of nitroxide compounds.

## CONCLUSIONS

TEMPO partitioning in the membranes of healthy and cancer cells of human lung tissues indicates that compared to healthy cells, the partition coefficients for the cancer cells are significantly higher. A positive correlation is observed between the temperature and the partition coefficient values for both cell types. The DG*K* values determined for TEMPO suggest that, compared to healthy cells, cancer cells more readily incorporate TEMPO molecules into their membrane. The lowest free energy change required to transfer TEMPO from an aqueous to a lipid phase of the membrane was observed in cancer cells at pH 7.3. Considering TEMPO as an anti-cancer drug for various types of cancer, in addition to standard chemotherapy, complementary alkalization therapy to change the acidic microenvironment to a slightly more alkaline one could be beneficial to some cancer patients. The TEMPO partitioning experiments described above were performed on four additional lung cancer patients. The characteristics of TEMPO partitioning were similar in all cases. However, the difference between the values of the TEMPO partitioning coefficients for lung normal and cancer cells varied and was case-dependent. The benefit derived from hyperthermia and/or alkalization may not be effective in all cases. Therefore, characterization of cells by TEMPO partitioning could be a valuable tool for choosing a proficient strategy for personalized cancer chemotherapy. Our experiments with C12SL indicate that the increased membrane dynamics in cancer cells could be a mechanism of enhanced partitioning of TEMPO.

## Abbreviations

NSCLC: non-small cell lung cancer
FAS: fatty acid synthase
SCD1: stearoyl-CoA desaturase 1
EPR: Electron Paramagnetic Resonance
